# Is Urinary NGAL Determination Useful for Monitoring Kidney Function and Assessment of Cardiovascular Disease? A 12-Month Observation of Patients with Type 2 Diabetes

**DOI:** 10.1155/2016/8489543

**Published:** 2016-12-05

**Authors:** Agnieszka Żyłka, Agnieszka Gala-Błądzińska, Paulina Dumnicka, Piotr Ceranowicz, Marek Kuźniewski, Krzysztof Gil, Rafał Olszanecki, Beata Kuśnierz-Cabala

**Affiliations:** ^1^St. Queen Jadwiga Clinical District Hospital No. 2, Rzeszow, Poland; ^2^Department of Medical Diagnostics, Faculty of Pharmacy, Jagiellonian University Medical College, Krakow, Poland; ^3^Department of Physiology, Jagiellonian University Medical College, Krakow, Poland; ^4^Department of Nephrology, Jagiellonian University Medical College, Krakow, Poland; ^5^Department of Pathophysiology, Faculty of Medicine, Jagiellonian University Medical College, Krakow, Poland; ^6^Department of Pharmacology, Faculty of Medicine, Jagiellonian University Medical College, Krakow, Poland; ^7^Department of Diagnostics, Chair of Clinical Biochemistry, Jagiellonian University Medical College, Krakow, Poland

## Abstract

*Background*. Diabetic kidney disease (DKD) may start as glomerular or tubular damage. We assessed kidney function during one-year-long observation of patients with type 2 diabetes mellitus (T2DM) after initiation of nephroprotective treatment, with emphasis on the changes in urinary neutrophil gelatinase-associated lipocalin (uNGAL), and evaluated the association between tubular damage and cardiovascular complications of T2DM.* Materials and Methods*. Adult T2DM patients (55) were assessed initially and 30 patients after 1 year. Albumin and uNGAL and creatinine were measured in first morning urine. Albumin/creatinine (uACR) and uNGAL/creatinine (uNCR) ratios were calculated.* Results*. In logistic regression, both uACR above 30 mg/g and uNCR the median (21.3 *μ*g/g) were associated with cardiovascular complications, independently of classical risk factors and diabetes duration. One year after initiation of treatment, a significant reduction in HbA_1c_ was observed. BMI and lipid profiles did not change. Increase in serum creatinine and reduction in eGFR occurred, along with decrease in uNGAL and uNCR. Increasing uNCR and uACR were associated with higher control HbA_1c_. The increase in uNCR was more frequent in patients with hypertension.* Conclusions*. Better glycemic control in T2DM patients results in improved tubular function, as reflected by reduced uNCR and uNGAL. First morning urine uNGAL and uNCR may be useful to assess renal function and cardiovascular risk, along with albuminuria and eGFR.

## 1. Introduction

The prevalence of diabetes worldwide is over 9 percent, and it is gradually increasing [1]. The World Health Organization (WHO) estimates that, in highly developed countries, 85 percent of population will suffer from type 2 diabetes mellitus (T2DM) [2]. The mortality due to diabetes complications, resulting from diabetic macroangiopathy, microangiopathy, or neuropathy, is an important social and clinical issue. In accordance with the latest 2016 European Society of Cardiology (ESC) guidelines, diabetic patients are considered to be at a very high or high risk of developing cardiovascular disease (CVD) [3]. It is assumed that 30–35 percent of genetically predestined individuals, receiving no or inadequate treatment, may develop angiopathy in renal microcirculation and diabetic kidney disease [4]. Glomerular filtration rate (GFR) lower than 60 mL/min/1.73 m^2^ further increases the risk of developing CVD [3]. Available studies clearly point to increased mortality rate of patients suffering from T2DM associated with nephropathy. However, early recognition of T2DM and adequate treatment inhibit progression of changes in blood vessels and contribute to favorable prognosis [3].

Kidney diseases are diagnosed on the basis of serum creatinine concentration and estimated GFR (eGFR), albuminuria, renal imaging, and histology following renal biopsy. However, low accuracy or invasiveness of these tests causes the fact that they frequently do not meet expectations of clinicians. In accordance with American Diabetes Association (ADA) 2016 standards, in order to detect or assess DKD, urinary albumin excretion and eGFR have to be measured annually [4]. Taking into account a limited diagnostic value of eGFR in detection of early renal dysfunction [5], as well as the fact that not all people with DKD have increased albuminuria, a search for new markers for kidney damage seems necessary. The new markers should be characterized by higher diagnostic sensitivity and specificity and should allow detecting the nonglomerular kidney damage [6–8].

Neutrophil gelatinase-associated lipokalin (NGAL), a member of the lipocalin protein family, has been recognized as one of the most promising biomarkers of early stages of kidney damage. It is a secreted protein with a molecular weight of 25 kDa, found in the neutrophil granules [9]. Recent studies focus mainly on the role of NGAL as a biomarker of acute kidney injury (AKI) [10, 11]. However, NGAL may also serve as a marker of chronic kidney disease (CKD), including DKD [12, 13]. Due to high biological variability of urinary NGAL (uNGAL), in CKD patients, uNGAL measurements should be accompanied by the assessment of urine creatinine concentration and calculation of uNGAL/creatinine ratio (uNCR) [14]. Prospective studies in DKD patients indicated the association between increasing uNGAL and the progression of kidney disease [15, 16] and negative correlation between uNCR and eGFR [16, 17], irrespective of albuminuria, although a positive correlation with albuminuria was also observed in some studies [16]. These observations in T2DM patients encourage the hypothesis that NGAL may be an earlier biomarker of DKD than albuminuria. The measurements of initial values of uNGAL and uNCR, and subsequent regular monitoring of their changes, seem to be useful in the assessment of kidney function in T2DM patients. Promising preliminary results were also obtained in our studies: namely, we found that uNCR exceeding 21.3 *μ*g/g may be useful for early prediction of renal tubular damage in the course of DKD [18].

The aim of the study was to assess the changes in renal function of T2DM patients during a 12-month observation, following the introduction of nephroprotective treatment according to ADA 2016 standards. Special attention was paid to the changes in uNGAL concentrations and uNCR values after treatment. We evaluated the correlations between the changes in markers of kidney function after a year-long treatment and the diabetes duration, blood glucose concentrations, and the use of medications affecting the renin-angiotensin-aldosterone system (RAAS) and the lipid metabolism. Additionally, the relationship between uACR and uNCR values and cardiovascular complications were assessed at the beginning of the study.

## 2. Materials and Methods

The study group consisted of women and men diagnosed with T2DM and undergoing treatment at the Department of Nephrology at St. Queen Jadwiga Clinical District Hospital No. 2 in Rzeszow, Poland. The inclusion criteria were age above 18 years, T2DM, and eGFR ≥60 mL/min/1.73 m^2^. The exclusion criteria were anemia, overt proteinuria or urinary albumin/creatinine ratio (uACR) > 300 mg/g, hematologic malignancies, systemic connective tissue diseases, allergies, infections, uncontrolled hypertension, and treatment with potentially nephrotoxic medications. Patients gave written informed consent for the study. The protocol received permission from the Bioethics Committee of the Regional Medical Chamber in Rzeszow, Poland (approval number 70/2014/B issued on 19 September 2014).

A cross-sectional analysis was conducted using data obtained at the first visit of patients. A prospective, observational study was conducted in patients with available follow-up data. At the beginning of the study patients underwent a careful clinical examination, including the assessment of body mass index (BMI) and blood pressure, as well as laboratory tests. Cardiovascular complications of T2DM were diagnosed in patients with ischemic heart disease, heart failure, systemic atherosclerosis, or ischemic stroke that occurred after the diagnosis of T2DM. In accordance with current clinical regulations [19], nephroprotective treatment was initiated, hypertension treatment was modified taking into account documented, outpatient blood pressure readings, diabetes treatment was adjusted taking into account outpatient measurements of glycemic status and the measurements of glycated hemoglobin (HbA_1c_), fluid and electrolyte balance was regulated, and hypolipemizing treatment was initiated or modified in order to control the lipid profile and the liver function parameters. Twelve months after the initiation of the study a subsequent clinical assessment of patients and laboratory tests were conducted.

Laboratory tests included fasting serum glucose, HbA_1c_, complete blood count, triglycerides, total cholesterol, low-density lipoprotein- (LDL-) and high-density lipoprotein- (HDL-) cholesterol, and serum creatinine. eGFR was calculated on the basis of CKD-EPI equation [20]. The urine tests included examination of the sediment, concentrations of uNGAL, albumin, and creatinine in first morning urine sample. uNGAL was measured using CMIA (chemiluminescent microparticle immunoassay), on the immunochemistry platform ARCHITECT® (ARCHITECT Analyzer, Abbott Diagnostics, Abbott Park, USA). uNCR and uACR were calculated. The laboratory tests were performed at the Department of Laboratory Diagnostics at St. Queen Jadwiga Clinical District Hospital No. 2 in Rzeszow (Poland) at the day of blood collection.

### 2.1. Statistical Analysis

Numbers of patients (percentages of the study group) were reported for categories. Mean ± standard deviation or median (upper-lower quartile) was reported for quantitative variables (depending on distribution as evaluated with Shapiro-Wilk's test). Chi-squared test was used to analyze contingency tables. The parametric tests were used for normally distributed data, and nonparametric tests were used for nonnormally distributed data. In detail, the results obtained at the beginning of the study and after 12 months were compared using paired *t*-test or Wilcoxon test. The differences between groups were tested with unpaired *t*-test or Mann–Whitney test. Correlations were assessed using Pearson's or Spearman's correlation coefficients. In particular, the correlations between changes in the markers of kidney function were evaluated; the change in the value of a given marker was defined as the difference between the control value (value after 12 months of treatment) and the initial value (at the beginning of the study). Multiple logistic regression was calculated to study the association between cardiovascular complications and uACR and uNCR values, with adjustment for classical cardiovascular risk factors. Results were considered statistically significant at *p* < 0.05. Statistica 12 (StatSoft, Tulsa, USA) software was used for computations.

## 3. Results

Initially, 55 patients were qualified for the study. Nineteen of them were diagnosed with cardiovascular complications of T2DM. Patients with cardiovascular complications were characterized with older age, higher albuminuria and uACR values, and higher uNCR values (Table 1). Median uNCR value in the group of 55 T2DM patients was 21.3 *μ*g/g. In multiple logistic regression, both uACR above 30 mg/g and uNCR the median were associated with cardiovascular complications independently of classical cardiovascular risk factors and diabetes duration (Table 2).

The follow-up data after 12 months from initiation of nephroprotective treatment were available for 30 patients. The prospective study group included 17 women (56%) and 13 men (44%), aged 64 ± 13 years. The median duration of T2DM at the beginning of the study was 9 (2–11) years. At the beginning of the study most patients were diagnosed with comorbidities: hypertension in 23 (77%), ischemic heart disease in 17 patients (31%), including one with the history of non-ST elevation myocardial infarction, systemic atherosclerosis in 5 (9%), and heart failure in 6 (11%) patients. Additionally, one patient had history of transient ischemic attack. No new cardiovascular complications were diagnosed during the follow-up. Most patients with hypertension received medications affecting the RAAS (angiotensin converting enzyme inhibitors or angiotensin receptor blockers). These medications were used by 21 patients (70%) at the beginning of the study. Only in 2 patients with hypertension, because intolerance was not treated by the RAAS inhibitors, one of these patients was repeatedly prone to develop hyperkalemia, whereas the other developed hypotension when RAAS inhibitors were added to alpha-blocker used because of urological disorder. During the study, 28 patients (93%) were treated with RAAS inhibitors. Twelve patients (40%) were treated with statins.

The characteristics of the study group at the beginning of the study and after 12 months are presented in Table 3. At the beginning of the study 26 patients (87%) had a BMI ≥25 kg/m^2^, and the percentage remained the same after 12 months. During the study, BMI decreased in 16 patients (53%), increased in 9 (30%), and did not change in the remaining 5 patients (17%). Mean HbA_1c_ decreased after 12 months of treatment (Table 1), and in 19 patients (63%) good glycemic control was achieved (HbA_1c_ < 6.5%). The treatment had no influence on the concentrations of total cholesterol, HDL- and LDL-cholesterol, or triglycerides (Table 3).

**Figure 1:**
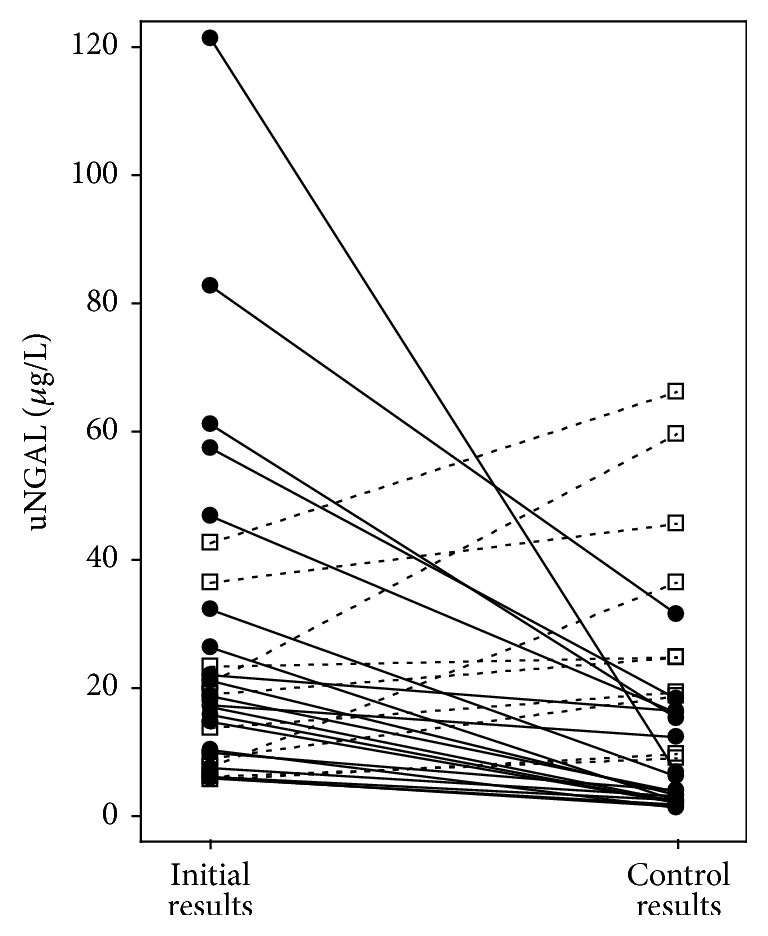
Urinary NGAL concentrations at the beginning of the study (initial results) and after 1 year of nephroprotective treatment (control results) among 30 DKD patients with available follow-up data. Closed circles and solid lines represent patients with decreasing uNGAL; open squares and dashed lines represent patients with increasing uNGAL. For abbreviations, see Table 1.

After 12 months, serum creatinine increased in 17 patients (57%) along with a decrease in eGFR (by 7 mL/min/1.73 m^2^ on average and 16 maximum). In 4 patients (13%) eGFR did not change, whereas in 9 (30%) it increased (by 4 mL/min/1.73 m^2^ on average and 10 maximum). Overall, average creatinine concentrations slightly increased, and average eGFR values in the whole study group decreased (Table 3). Simultaneously, average concentrations of uNGAL and urinary albumin decreased; however, in the case of albuminuria and uACR the difference was not statistically significant (Table 3). After a year of treatment, uNGAL concentrations increased in 10 patients (33%), but in 20 patients (67%) there was a considerable decrease in uNGAL (Figure 1). In turn, uNCR increased in 9 patients (30%) and decreased in 21 (70%). Albuminuria increased in 12 (40%) and uACR increased in 11 (73%), while it decreased in the remaining patients.

**Figure 2:**
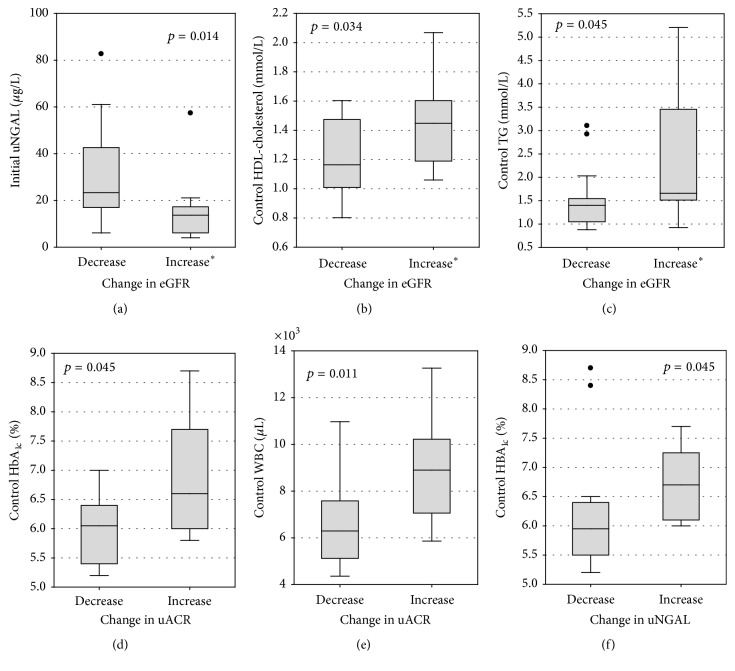
Statistically significant differences in laboratory test results between patients with different direction of change in the studied markers of kidney function. The change in the marker of kidney function was defined as the difference between the control value (after 12 months of treatment) and the initial value (at the beginning of the study). ^*∗*^Increase or no change in eGFR. TG, triglycerides; see Tables 1 and 3.

**Figure 3:**
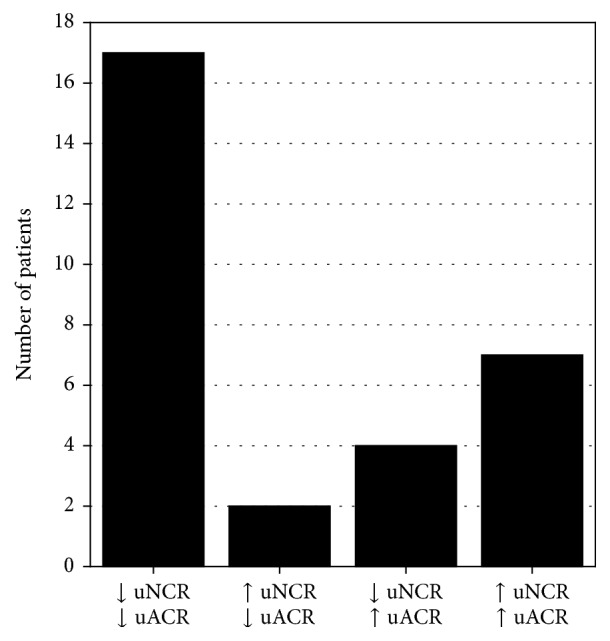
Numbers of patients in whom either a decrease or an increase in the values of uNCR and uACR was observed after 12 months of treatment in comparison with initial values. ↓: decrease in the value of a given marker as assessed after 12 months of the study; ↑: increase in the value of a given marker as assessed after 12 months of the study; For abbreviations, see Table 1.

Patients whose eGFR values decreased during the study had higher initial concentrations of uNGAL and lower control concentrations of HDL-cholesterol and of triglycerides than patients whose eGFR values did not change or increased (Figures 2(a)–2(c)). The change in eGFR (defined as the difference between eGFR values at the end of the study and eGFR values at the beginning) was positively correlated with control concentrations of total cholesterol (*R* = 0.43; *p* = 0.022) and the initial and control concentrations of HDL-cholesterol (*R* = 0.39; *p* = 0.042 and *R* = 0.48; *p* = 0.010, resp.). The change in eGFR was negatively correlated with the initial values of uNCR (*R* = −0.38; *p* = 0.036) and the control concentration of uNGAL (*R* = −0.51; *p* = 0.004). The differences between the concentrations of uNGAL after 12 months and the initial concentrations correlated with, analogically assessed, changes in the concentrations of urinary albumin (*R* = 0.42; *p* = 0.026). A similar correlation was observed between the changes in uNCR and uACR (*R* = 0.48; *p* = 0.011). In most patients, a decrease in the values of both markers was observed during the study (Figure 3). In comparison with patients whose uACR values decreased, patients whose uACR values increased during 12 months had higher control values of HbA_1_ and higher leukocyte count (Figures 2(d) and 2(e)). The increase in uNGAL concentrations was associated with higher control concentrations of HbA_1c_ (Figure 2(f)). Additionally, diabetes duration correlated positively with the changes in albuminuria (*R* = 0.40; *p* = 0.033) and uNCR (*R* = 0.46; *p* = 0.010).

The increase in uNCR was significantly more frequent (*p* = 0.048) in hypertensive patients (9 out of 23 patients, 45%) than in patients with no hypertension (0 out of 7 patients). No correlations were observed between the increase or decrease in the values of kidney function markers and the presence of other comorbidities or medications applied. Furthermore, no correlations were observed between the changes in the values of kidney function markers and the age or gender of patients.

## 4. Discussion

DKD remains one of the most serious complications of diabetes. Its late recognition and inadequate treatment may lead to end-stage renal disease and the need for renal replacement therapy. However, although DKD is progressive and irreversible, there are studies indicating that early recognition of the disease and initiation of nephroprotective treatment may slow down its progression.

In T2DM patients, kidney function must be evaluated in a comprehensive manner. The evaluation should take into account not only GFR and albuminuria indicating the possible damage to the filtration membrane, but also the function of renal tubules. To assess the function of renal tubules, we analyzed the changes in uNGAL and uNCR measured in a first morning urine sample during a 12-month observation of patients suffering from T2DM and CKD stages G1 and G2, with accompanying normal or moderately increased albuminuria (A1 or A2), according to KDIGO criteria [20].

Patients recruited for our study had medical conditions and symptoms typical for the T2DM related metabolic syndrome and insulin resistance. These was excess weight or obesity (more than 78% of patients), hypertension (more than 77% of patients), or dyslipidemia requiring drug treatment (40% of patients). Taking into account cardiovascular disease observed in the ARETAEUS1 study, a study examining the clinical profile of the Polish population with T2DM of short duration, the patient group in our study was a representative sample [21].

According to the latest ADA 2016 and ESC 2016 guidelines, T2DM patients should change their dietary habits, maintain regular physical activity, and reduce their body weight [19]. Most patients in our study group were able to lose weight due to pharmacological treatment, as well as changes in diet and lifestyle.

According to the SHARP study, in order to reduce the risk of CVD in CKD patients, statins should be used [22]. Moreover, statins play a significant role in nephroprotective treatments [23]. In our study, 40% of patients were treated with statins. In accordance with ESC 2016 guidelines, none of patients was treated with fibrates, even though hypertriglyceridemia is a most pronounced lipid disorder in T2DM patients [24].

From the clinical point of view, it is important to observe that in most patients glycemic control, measured with HbA_1c_, improved after 12 months. This, in turn, had probably a direct influence on the reduction of albuminuria and the uACR values. Albuminuria is a recognized risk factor both for the progression of DKD and for cardiovascular disease [3]. Consequently, for nephrologists, reduction of albuminuria is crucial in the treatment of T2DM [19]. However, it has to be remembered that increased albuminuria (30–300 mg/g) is the first indicator of DKD only in a part of T2DM patients. In about 30% of DKD patients, progressive reduction in GFR is not accompanied by increased excretion of urinary albumin [25].

Since the assessment of kidney function based on eGFR and albuminuria is far from being satisfactory, and it allows only monitoring the function of the glomerular filtration membrane, we have made an attempt to assess the kidney function in T2DM patients with respect to possible damage to renal interstitium, by measuring uNGAL. uNGAL excretion increases significantly in the epithelial cells of the ascending limb of the loop of Henle in renal tubules in a response to ischemia or toxins [26]. It is assumed that NGAL may play a significant role in pathophysiology of kidney adaptation to the destructive influence of diabetic environment on renal tubules [14].

In this study, following the implemented treatment, a statistically significant decrease in uNGAL and uNCR was obtained after 12 months. The observed tendency for decreasing uNGAL and uNCR values may be a confirmation of the positive influence of multifactorial treatment of T2DM patients, aimed at inhibiting the progression of renal tubules damage. During a 3.5-year observation of T2DM patients, Nielsen et al. [27] observed a positive correlation of uNCR with HbA_1c_. Similarly, we observed a decrease in HbA_1c_ during a 12-month study, accompanied by a decrease in tubular proteinuria (uNCR values).

The observed significant decrease in eGFR confirms the observation that in people over the age of 30, the decline in GFR values is a physiological process (the annual rate of decrease in GFR is 0.75–1 mL/min/1.73 m^2^) [28, 29], further accelerated to 2.3–5.4 mL/min/1.73 m^2^ in DKD [30]. In our study, the decrease in eGFR values may also be a consequence of blocking the RAAS in most patients. Such treatment aims at lowering the glomerular filtration pressure, which in the initial phase of DKD leads also to a clinically insignificant decrease in eGFR. However, when applied for a longer time, such treatment has nephroprotective and cardioprotective effects, independently of blood pressure values [3]. According to ADA standards, all patients with uACR greater than 30 mg/g, irrespective of eGFR values, should be treated with angiotensin converting enzyme inhibitors (ACEI) and angiotensin II receptor blockers. In such patients, it is necessary to monitor the serum creatinine and kalemia. This treatment is not recommended in primary prevention of DKD with normotension, normal range of albuminuria, and normal eGFR [19]. Treatment with ACEI leads to the reduction in hemoglobin concentration [31, 32]. In this study the decrease in hemoglobin concentrations (statistically significant, but clinically insignificant), confirms, together with the hypotensive effect, the effectiveness of ACEI.

Other authors, in 12-month follow-up of DKD patients, observed the increase in uNGAL [33], as well as inverse correlations between uNGAL, uACR, and GFR in T2DM patients [15–17], which is contrary to our results. The discrepancies may result from different patient care and from racial differences in populations studied (all patients in our study were of Caucasian race).

The authors of ESC 2016 guidelines stress the potential benefits of introducing novel urinary biomarkers in selected patient populations, as these may contribute to improved assessment of cardiovascular risk [3]. Clinical trials aimed at finding the optimal biomarker pass through successive phases [34]. The authors of this study have observed that, at the time of the first visit, patients with cardiovascular disease had higher values of uACR and uNCR than patients without cardiovascular disease. Both uACR and uNCR were independently associated with cardiovascular complications, irrespective of classical cardiovascular risk factors. No new incidents of cardiovascular disease were diagnosed in the study group during the 12-month follow-up. During this time significant decrease in both uNGAL and uNCR and no increase in albuminuria were observed. Our results indicate that renal complications of T2DM, both those involving glomeruli and tubules, are significantly associated with cardiovascular complications of diabetes. Thus, the measurement of uNCR may be helpful in the clinical prediction of CVD in T2DM patients.

The major limitation of our study is the small number of patients enrolled. Therefore, we cannot draw any definitive conclusions. However, the results seem promising and should be validated in larger studies.

## 5. Conclusions

On the basis of a 12-month observation of early-phase DKD patients it can be concluded that multifactorial nephroprotective treatment, focused primarily on the improvement of glycemic control, has a positive effect on the function of renal tubules as reflected by the diminishing concentrations of uNGAL and uNCR. Additionally, uNCR may be considered as independent predictor of the increased risk of CVD in the population studied.

## Figures and Tables

**Table 1:** Baseline characteristics of the studied group of 55 T2DM patients with respect to cardiovascular complications of diabetes.

	Patients with cardiovascular complications (*N* = 19)	Patients without cardiovascular complications (*N* = 36)	*p*
Age, years	70 ± 11	59 ± 15	0.022
Female sex	10 (53)	19 (53)	1.0 ^NS^
Ischemic heart disease, *N* (%)	17 (89)	—	—
Heart failure, *N* (%)	6 (32)	—	—
Systemic atherosclerosis, *N* (%)	5 (26)	—	—
T2DM duration, years	7 (4–10)	5 (1–10)	0.1 ^NS^
Hypertension, *N* (%)	16 (84)	26 (72)	0.3 ^NS^
Dyslipidemia, *N* (%)	18 (95)	33 (92)	0.7 ^NS^
BMI, kg/m^2^	32.6 ± 7.6	30.8 ± 5.2	0.5 ^NS^
HbA_1c_, %	6.2 (6.1–6.6)	7.5 (6.2–9.4)	0.2 ^NS^
WBC, 10^3^/*μ*L	8.0 (5.3–9.7)	7.1 (5.9–8.4)	0.8 ^NS^
Serum creatinine, *μ*mol/L	64.5 (60.1–82.2)	65.8 (58.8–76.9)	0.6 ^NS^
eGFR, mL/min/1.73 m^2^	87 (71–94)	95 (81–99)	0.07 ^NS^
Albuminuria, mg/L	12.3 (7.2–41.2)	9.5 (6.3–13.4)	0.048
uACR, mg/g	16.0 (7.5–53.6)	7.8 (3.4–13.2)	0.005
uNGAL, *μ*g/L	22.3 (10.4–56.9)	18.8 (8.8–42.6)	0.7 ^NS^
uNCR, *μ*g/g	29.1 (13.4–58.8)	16.2 (9.5–38.8)	0.038

T2DM, type 2 diabetes mellitus; *N*, number of patients; BMI, body mass index; HbA_1c_, hemoglobin A_1c_; WBC, white blood cells; eGFR, estimated glomerular filtration rate; uNGAL, urine neutrophil gelatinase-associated lipocalin; uNCR, urinary NGAL/creatinine ratio; uACR, urinary albumin/creatinine ratio; NS, nonsignificant result.

**Table 2:** Multiple logistic regression model showing the association between selected variables and cardiovascular complications among 55 T2DM patients evaluated at the beginning of the study.

Independent variables	Odds ratio (95% confidence interval)	*p*
Age, years	1.11 (0.99–1.25)	0.07 ^NS^
Female sex	0.44 (0.05–3.80)	0.4 ^NS^
T2DM duration, years	1.03 (0.84–1.26)	0.8 ^NS^
BMI, kg/m^2^	1.22 (1.00–1.48)	0.041
Hypertension	5.21 (0.15–185)	0.3 ^NS^
Dyslipidemia	0.16 (0.02–1.68)	0.1 ^NS^
uACR > 30 mg/g	25.20 (1.01–639)	0.042
uNCR > 21.3 *μ*g/g	14.99 (1.01–247)	0.048
Whole model	chi^2^ = 19.8; *p* = 0.011	

For abbreviations, see Table 1.

**Table 3:** Characteristics of 30 T2DM patients with available follow-up data at the beginning of the study (baseline results) and after 12-month follow-up (control results).

	Baseline results	Control results	*p*
BMI, kg/m^2^	30.9 ± 5.5	31.4 ± 5.7	0.6 ^NS^
HbA_1c_, %	7.98 ± 1.99	6.31 ± 0.93	0.037
Hemoglobin, g/dL	14.2 ± 1.4	13.9 ± 1.4	0.019
WBC, 10^3^/*μ*L	7.59 ± 2.39	7.34 ± 2.67	0.5 ^NS^
Total cholesterol, mmol/L	5.38 (4.11–5.95)	4.78 (4.01–5.86)	0.3 ^NS^
LDL-cholesterol, mmol/L	3.10 (2.07–3.75)	2.62 (1.98–3.94)	0.3 ^NS^
HDL-cholesterol, mmol/L	1.32 (1.01–1.53)	1.36 (1.03–1.49)	0.1 ^NS^
Triglycerides, mmol/L	1.57 (1.13–1.89)	1.53 (1.16–2.03)	0.5 ^NS^
Serum creatinine, *μ*mol/L	68.1 (60.1–76.9)	69.0 (61.9–77.8)	0.035
eGFR, mL/min/1.73 m^2^	94.4 (79.7–98.3)	87.0 (74.6–99.0)	0.023
Albuminuria, mg/L	8.53 (6.59–13.53)	5.55 (2.14–19.75)	0.2 ^NS^
uACR, mg/g	7.49 (3.39–13.38)	4.69 (2.86–43.41)	0.1 ^NS^
uNGAL, *μ*g/L	18.00 (9.00–32.20)	9.35 (2.50–19.30)	0.018
uNCR, *μ*g/g	16.18 (10.00–33.72)	8.82 (3.09–26.83)	0.037
Leukocyturia, *n*/(%)	3 (10)	5 (17)	0.4 ^NS^

LDL, low-density lipoprotein; HDL, high-density lipoprotein; see Table 1.
